# 2′-Fucosyllactose Attenuates *Fusobacterium nucleatum* Virulence and Modulates the Oral Microbiota

**DOI:** 10.3390/microorganisms14071603

**Published:** 2026-07-22

**Authors:** Xinyu Wu, Shuangshuang Han, Yifeng Wang, Xintong Chen, Sijia Liu, Mengxiang Li, Shan Lin, Liying Feng, Xiaoya Guo, Zhengang Li, Huilin Hao, Xin Wang, Di Huang, Lu Feng, Bin Liu, Lei Wang

**Affiliations:** 1 National Key Laboratory of Intelligent Tracking and Forecasting for Infectious Diseases, TEDA Institute of Biological Sciences and Biotechnology, Nankai University, Tianjin 300457, China; 1120230813@mail.nankai.edu.cn (X.W.); 2120241526@mail.nankai.edu.cn (S.H.); yfwnk@mail.nankai.edu.cn (Y.W.); cxtchenxinxin@outlook.com (X.C.); sijialiu012@163.com (S.L.); l18438596263@126.com (M.L.); lins@mail.nankai.edu.cn (S.L.); 13920014004@163.com (L.F.); 18834820179@163.com (X.G.); lizhengang977@126.com (Z.L.); haohuilin@mail.nankai.edu.cn (H.H.); wangxin021024@163.com (X.W.); huangdi@nankai.edu.cn (D.H.); fenglu63@nankai.edu.cn (L.F.); 2 Key Laboratory of Molecular Microbiology and Technology, Nankai University, Tianjin 300457, China; 3 Nankai International Advanced Research Institute, Nankai University Shenzhen, Shenzhen 518045, China; 4 Southwest United Graduate School, Kunming 650092, China

**Keywords:** 2′-Fucosyllactose, *Fusobacterium nucleatum*, attenuation of virulence, human gingival epithelial cell, oral microbiota

## Abstract

*Fusobacterium nucleatum* (*F. nucleatum*) is a key periodontal pathobiont associated with oral inflammation. This bacterium forms biofilms and expresses adhesins that facilitate its adhesion to and invasion of gingival epithelial cells. These processes disrupt the epithelial barrier and trigger oral inflammation, and in some cases, systemic inflammation. Conventional antimicrobial strategies predominantly depend on the utilization of antibiotics. Nevertheless, this can result in the proliferation of drug-resistant strains and the disruption of the oral microbiome equilibrium. As the predominant human milk oligosaccharide, 2′-Fucosyllactose (2′-FL) demonstrates considerable promise in inhibiting pathogenic bacterial adhesion and fortifying epithelial barrier function, mediated by its characteristic structural and bioactive attributes. In this study, we showed that 2′-FL attenuates the expression of virulence genes in *F. nucleatum*, reduces biofilm formation, and suppresses the bacterium’s ability to adhere to human gingival epithelial cells (HGECs). Furthermore, at the transcriptional level, 2′-FL suppressed *F. nucleatum*-induced inflammatory cytokine overexpression in both HGECs and RAW 264.7 macrophages, and upregulated barrier-related proteins (ZO-1, Occludin) and MUC-1 gene expression in HGECs. In vivo studies demonstrated the inhibitory effect of 2′-FL on *F. nucleatum*-induced periodontal injury in Balb/c mice. Furthermore, 16S rRNA sequencing analysis demonstrated that 2′-FL modulated oral microbiota composition of healthy volunteers and significantly reduced the abundance of *Fusobacterium*.

## 1. Introduction

*Fusobacterium nucleatum* is a Gram-negative opportunistic pathogen. It has the capacity to contribute to various oral diseases, including halitosis, dental plaque, dental caries, periodontitis and oral cancer [1]. Beyond its oral pathogenicity, mounting evidence has implicated *F. nucleatum* in extra-oral systemic diseases, including colorectal cancer, adverse pregnancy outcomes, cardiovascular disorders, and Alzheimer’s disease, primarily through hematogenous dissemination, immune modulation, and metabolic reprogramming [2]. As a keystone species within oral biofilms, *F. nucleatum* orchestrates dysbiotic microbial communities by coaggregating with both early and late colonizers, thereby facilitating the transition from symbiotic to pathogenic states in subgingival plaque [3]. *F. nucleatum* expresses a repertoire of virulence factors, including the FadA adhesin, the Fap2 lectin and lipopolysaccharide (LPS), amongst others, which collectively facilitate biofilm formation, adherence to and invasion of oral epithelial cells, immune evasion, and the induction of pro-inflammatory responses [4,5]. Therefore, effective suppression of *F. nucleatum* virulence can attenuate its damage to host oral tissues, thereby preventing oral diseases and maintaining systemic health.

The global escalation of antimicrobial resistance (AMR), driven by the widespread and often indiscriminate use of conventional antibiotics and antimicrobial agents in oral healthcare, has severely constrained therapeutic efficacy against oral pathogens [6]. Consequently, there is an urgent need to develop alternative anti-infective strategies. In recent years, research interest has increasingly focused on natural bioactive compounds, including plant-derived polyphenols [7], antimicrobial peptides [8], prebiotics [9], and probiotic metabolites [10], which are considered promising anti-pathogenic candidates against *F. nucleatum*. Unlike traditional antibiotics, which often exert strong bactericidal pressure and promote the emergence of resistant mutants, these natural agents typically act through multi-target mechanisms, including the disruption of bacterial biofilm architecture, attenuation of virulence factor expression, modulation of oral microbiota dysbiosis, and enhancement of host immune responses, while imposing relatively low selective pressure for resistance development.

Human milk oligosaccharides (HMOs) are naturally occurring bioactive constituents endogenous to human milk, representing the third most abundant solid nutrient component following lactose and lipids in human milk [11]. As non-digestible oligosaccharides, HMOs have been demonstrated to orchestrate systemic immunometabolic homeostasis by modulating host immune responses, enhancing metabolic regulation, antagonizing pathogen adhesion, and sculpting the composition and functional capacity of the gut microbiota [12,13]. 2′-Fucosyllactose (2′-FL), the most abundant HMO in human milk, has garnered considerable attention for its prebiotic properties. Existing evidence indicates that 2′-FL functions as a molecular decoy for pathogenic adhesins, thereby inhibiting the attachment of enteric pathogens, including enterohemorrhagic *Escherichia coli* O157 and *Group B Streptococcus* (GBS), to host epithelial cells [14,15]. Additionally, 2′-FL promotes the abundance of *Bifidobacteria*, restores gut microbial homeostasis, ameliorates aging-associated osteoporosis and metabolic dysregulation, modulates host immunity, and alleviates inflammatory bowel disease (IBD) and irritable bowel syndrome (IBS) [16].

Despite these advances, the effects of 2′-FL on *F. nucleatum* and its regulatory role in oral microecology remain elusive. Herein, we investigated the impact of 2′-FL on *F. nucleatum* virulence and delineated the underlying mechanisms. Our findings, derived from in vitro assays and animal models, demonstrate that 2′-FL suppresses *F. nucleatum* biofilm formation, downregulates virulence gene expression, attenuates pathogen-induced gingival epithelial cell damage and pro-inflammatory cytokine production, and mitigates gingival tissue destruction in mice. Furthermore, intervention of 2′-FL in healthy volunteers significantly modulated oral microbiota composition and reduced the abundance of *Fusobacterium*. Collectively, these results underscore the considerable translational potential of 2′-FL in oral care product development for maintaining oral microecological health.

## 2. Materials and Methods

### 2.1. Bacterial Culture

*Fusobacterium nucleatum* (ATCC 25586) was purchased from Beijing Beina Chuanglian Institute of Biotechnology (BNCC, Beijing, China) and propagated under anaerobic conditions in a COY anaerobic chamber (80% N_2_, 10% H_2_, and 10% CO_2_) at 37 °C. For liquid cultivation, brain heart infusion (BHI) broth was supplemented with 0.5% hemin solution (1 mg/mL), 0.1% vitamin K_1_ solution (1 mg/mL), and 0.5 g/L L-cysteine hydrochloride. Solid medium was prepared by adding 5% defibrinated sheep blood and 2% agar to the aforementioned liquid medium.

### 2.2. Crystal Violet Staining for Biofilm Assay

*F. nucleatum* was inoculated at a density of 1 × 10^6^ CFU/mL into 96-well plates (100 μL per well) containing BHI liquid medium supplemented with 2′-FL, lacto-N-neotetraose (LNnT), fructooligosaccharides (FOS), and galactooligosaccharides (GOS) at final concentrations of 0.2% and 0.8% (*w*/*v*). BHI medium without any carbohydrate supplementation served as the control. After anaerobic incubation for 48 h, the bacterial suspension was removed, and the adherent cells were fixed with 95% methanol. The fixed cells were then stained with 0.1% (*w*/*v*) crystal violet for 10 min, followed by washing to remove unbound dye. Stained cells were visualized and photographed under a light microscope. Subsequently, the bound crystal violet was solubilized by adding 95% ethanol and incubating at 37 °C for 15 min, and the absorbance was measured at 590 nm (OD_590_). Based on the concentration screening experiments, 0.8% (*w*/*v*) 2′-FL was selected for subsequent studies because it produced the greatest reduction in biofilm formation while maintaining excellent cytocompatibility toward HGECs.

### 2.3. Scanning Electron Microscope (SEM)

After placing the sterile glass slide (φ = 14 mm) coated with artificial saliva into a 24-well plate, *F. nucleatum* suspension (1 × 10^6^ CFU/mL) was added into each well. For the treatment group, 2′-FL was simultaneously added to achieve a final concentration of 0.8% (*w*/*v*), whereas the control group received an equal volume of sterile PBS. After anaerobic incubation for 48 h, the bacterial solution was aspirated, washed three times with sterile PBS, fixed with 2.5% glutaraldehyde at 4 °C overnight, dehydrated, lyophilized, sprayed gold, and observed under scanning electron microscopy (SEM).

### 2.4. Cell Culture

Human gingival epithelial cells (HGECs) were purchased from Shanghai Enzyme-linked Biotechnology Co., Ltd. (Shanghai, China) and cultured in DMEM (Meilun, Tianjin, China) supplemented with 10% fetal bovine serum (Gibco, Waltham, MA, USA) and 1% penicillin/streptomycin (Beyotime, Shanghai, China) at 37 °C with 5% CO_2_ in an incubator (ThermoFisher, Waltham, MA, USA).

### 2.5. Adhesion of F. nucleatum to HGECs: Bacterial Enumeration and Fluorescence Labeling

HGECs were seeded into 12-well plates and infected with logarithmic-phase *F. nucleatum* at a multiplicity of infection (MOI) of 100. For the 2′-FL treatment group, 2′-FL was simultaneously added to achieve a final concentration of 0.8% (*w*/*v*). Following incubation, cells were washed three times with sterile PBS to remove non-adherent bacteria, lysed with 1% Triton X-100 at room temperature for 5 min, and serially diluted. The lysates were plated onto Columbia blood agar plates, and adherent bacteria were enumerated as CFU after anaerobic incubation.

For fluorescence labeling, *F. nucleatum* was stained with a fluorescein isothiocyanate (FITC) working solution. The bacterial suspension was vortexed, incubated in the dark for 30 min, and washed three times with PBS. FITC-labeled *F. nucleatum* was co-incubated with HGECs at 37 °C for 2 h. After washing twice with PBS, cells were fixed with 4% paraformaldehyde at room temperature for 20 min, rinsed three times with PBS (10 min per wash), and mounted with 50 μL of DAPI-containing antifade mounting medium. Images were acquired using a confocal laser scanning microscope (Leica, Wetzlar, Germany).

### 2.6. RNA Extraction and Quantitative Real-Time PCR (qRT-PCR)

Total bacterial RNA was extracted using TRIzol reagent combined with liquid nitrogen grinding and reverse-transcribed into complementary DNA (cDNA) using the TransScript kit (GeneStar, Beijing, China) according to the manufacturer’s instructions. The cDNA was stored at −20 °C until use. Quantitative real-time PCR was performed on an Applied Biosystems 7500 Real-Time PCR System (ThermoFisher, Waltham, MA, USA) using SYBR Green dye, and relative gene expression levels were calculated using the 2^−∆ΔCt^ method. The bacterial housekeeping gene 16S rRNA was used as the internal reference for normalization. The primers targeting *F. nucleatum* virulence genes are listed in Supplementary Table S1.

### 2.7. Molecular Docking of Adhesin Proteins

The three-dimensional structure of 2′-FL was retrieved from the PubChem database (https://pubchem.ncbi.nlm.nih.gov/). Protein structures of *F. nucleatum* were obtained from the RCSB Protein Data Bank (https://www.rcsb.org/) and the AlphaFold Database (https://alphafold.ebi.ac.uk/ accessed on 17 July 2026). Protein structures were pre-processed using the Prepare function in Molecular Operating Environment (MOE, version 2022.02). Molecular docking simulations were performed using the MOE Dock module. The top-ranked conformation was selected based on the lowest S score, and intermolecular interactions were analyzed. Three-dimensional docking visualizations were generated using PyMOL (version 3.1.6).

### 2.8. Detection of Inflammatory Cytokine and Barrier Protein Expression in HGECs

HGECs were seeded into 6-well plates at a density of 1 × 10^6^ cells per well. Upon reaching approximately 80% confluence, cells were infected with *F. nucleatum* (MOI = 100). For the 2′-FL treatment group, 2′-FL was added to a final concentration of 0.8% (*w*/*v*), with PBS-treated cells serving as the control. Following 12 h of co-culture, cells were harvested for RNA extraction and qRT-PCR analysis of IL-6, IL-8, IL-1β, and TNF-α mRNA expression levels. The targeted primers are listed in Supplementary Table S2.

### 2.9. Detection of Inflammatory Cytokine Expression in RAW264.7 Macrophages

RAW264.7 macrophages were seeded into 6-well plates and treated as described in method 8. After 24 h of co-culture, cells were collected for RNA extraction and qRT-PCR analysis of IL-6, IL-1β, and TNF-α mRNA expression. The targeted primers are listed in Supplementary Table S3.

### 2.10. Animal Experiments

All animal procedures were conducted in strict accordance with the Guide for the Care and Use of Laboratory Animals and were approved by the Institutional Animal Care and Use Committee (IACUC) of Nankai University (Tianjin, China) under protocol number [2026-SYDWLL-000159]. Male Balb/c mice (5 weeks old) were purchased from Vital River Laboratory Animal Technology Co., Ltd. (Beijing, China) and randomly divided into three groups, Control, *Fn*, and *Fn* + 2′-FL (n = 6 per group); *F. nucleatum* was administered via periodontal local injection at a dosage of 10 μL per mouse. For the *Fn* + 2′-FL group, the bacterial suspension was resuspended in 0.8% (*w*/*v*) 2′-FL solution prior to injection, and 200 μL of 0.8% (*w*/*v*) 2′-FL solution was topically applied to the oral cavity three times daily. After 14 days, oral samples were collected to quantify *F. nucleatum* colonization. The maxillae were subjected to micro-CT (micro computed tomography) imaging, and micro-CT scanning and morphometric analysis were conducted by an investigator fully blinded to the group assignments.

### 2.11. 16S rRNA Sequencing Analysis

In this pilot study, twenty Healthy volunteers of both genders, aged 18–50 years with good oral hygiene, no active periodontitis or untreated dental caries, and no systemic diseases were included. Current smokers and individuals who had used antibiotics, probiotics, or commercial mouthwashes within the past one month were strictly excluded. Participants were randomly assigned to the control or 2′-FL group using a computer-generated randomization sequence. Owing to the nature of the intervention, participant blinding was not feasible. However, microbiome analysis was performed using coded samples. Participants maintained their baseline dietary habits but were instructed to avoid any other functional foods, xylitol gums, or oral care supplements during the 14-day period. Compliance was monitored daily via a digital check-in log where participants recorded each rinsing session. All participants provided written informed consent before enrollment in the study. Participants in control group (rinsing with pure water, n = 8) or a treatment group (rinsing with 0.8% (*w*/*v*) 2′-FL aqueous solution, n = 10) were instructed to rinse with 15–20 mL of the assigned mouthwash for 30 s, three times daily (30 min after morning and evening tooth brushing and after lunch), and then expectorate the rinse without swallowing. Participants were asked to refrain from eating or drinking for at least 30 min following each rinsing session. After 14 days, dental plaque samples were collected for 16S rRNA gene sequencing to characterize microbial community composition. The V3–V4 hypervariable regions of the bacterial 16S rRNA gene were amplified using primer pairs 338F (5′-ACTCCTACGGGAGGCAGCAG-3′) and 806R (5′-GGACTACHVGGGTWTCTAAT-3′), followed by library preparation. Paired-end raw reads were quality-filtered using fastp (version 0.23.4) and assembled using FLASH (version 1.2.11) [17]. Operational taxonomic units (OTUs) were clustered at 97% sequence identity using USEARCH (version 11), and chimeric sequences were removed. Taxonomic classification was performed using the RDP Classifier (version 2.11) against the Silva 138.2/16S_bacteria database with a confidence threshold of 70%. Alpha- and beta-diversity indices were calculated using Mothur (version 1.30.2) and visualized using R (version 3.3.1). Principal coordinate analysis (PCoA) based on Bray–Curtis distances combined with analysis of similarities (ANOSIM) was employed to evaluate overall bacterial community differences among samples. Community bar plots were generated to illustrate the distribution of dominant taxa. Wilcoxon rank-sum tests were used for two-group comparisons, and linear discriminant analysis effect size (LEfSe) was performed to identify biomarker species contributing to group differentiation [18]. All analyses were conducted on the Majorbio Cloud Platform (https://v.majorbio.com).

### 2.12. Statistical Analysis

All experiments were repeated at least three times. Data were analyzed and graphed using GraphPad Prism (version 8.0.1; GraphPad Inc., San Diego, CA, USA). All data were expressed as the mean ± standard deviation (SD). Differences between two groups were evaluated using an unpaired two-tailed Student’s *t* test, whereas comparisons among multiple groups were analyzed by one-way analysis of variance (ANOVA) followed by Tukey’s multiple comparison tests. Statistical significance was defined as *p* < 0.05.

## 3. Results

### 3.1. 2′-FL Inhibits Biofilm Formation of F. nucleatum

**Figure 1:**
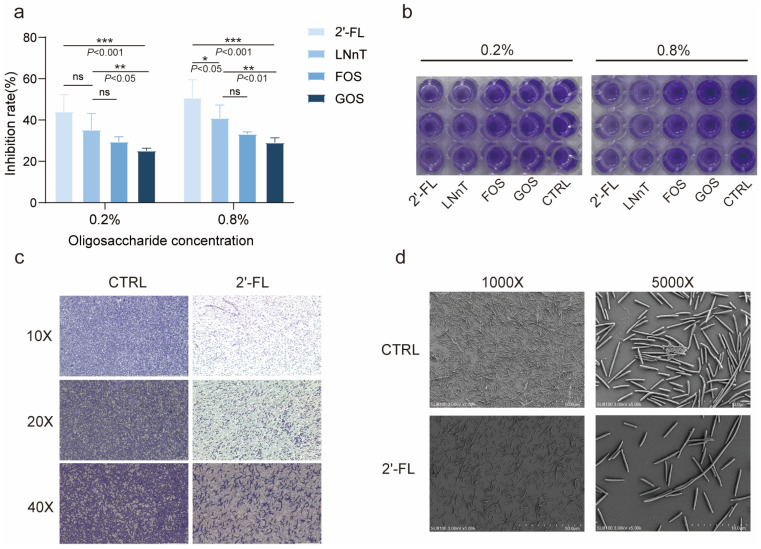
2′-FL inhibits *F. nucleatum* biofilm formation. (**a**) Quantitative analysis of biofilm biomass following 48 h co-incubation of *F. nucleatum* with 2′-FL, LNnT, FOS, or GOS at 0.2% or 0.8% (*w*/*v*). Data represent the percentage inhibition relative to the untreated control (n = 3). Statistical significance was determined by one-way ANOVA with Tukey’s multiple comparisons test. (* *p*  <  0.05, ** *p*  <  0.01, *** *p*  <  0.001, ns not significant) (**b**) Representative macroscopic images of stained biofilms in 96-well plates. (**c**) Light microscopy images of biofilms at 10×, 20×, and 40× magnifications. (**d**) Scanning electron micrographs illustrating biofilm ultrastructure at 1000× and 5000× magnifications. Scale bars are indicated.

Crystal violet staining is widely used to quantify total biofilm biomass, as the dye binds to bacterial cells and extracellular matrix components, including polysaccharides and proteins [19]. To evaluate the inhibitory effects of different oligosaccharides on *F. nucleatum* biofilm formation, the bacterium was co-incubated with 2′-FL, LNnT, FOS, or GOS at concentrations of 0.2% and 0.8% (*w*/*v*) in 96-well microtiter plates for 48 h, followed by crystal violet staining and quantification. As shown in Figure 1a, all oligosaccharides tested exhibited varying degrees of biofilm inhibition. At the lower concentration (0.2%), 2′-FL significantly reduced biofilm biomass and showed greater inhibitory activity than FOS and GOS (*p* < 0.05), although no statistically significant difference was observed between 2′-FL and LNnT. Upon increasing the concentration to 0.8%, the inhibitory efficacy of 2′-FL was markedly enhanced, resulting in a significantly higher suppression rate than those observed for LNnT (*p* < 0.05), FOS (*p* < 0.01), and GOS (*p* < 0.001). Consistent with the quantitative data, macroscopic examination of stained wells revealed dense, purple-colored biofilms in control and FOS/GOS-treated groups, whereas 2′-FL-treated wells, particularly at 0.8%, displayed visibly attenuated staining intensity (Figure 1b). Given the superior and concentration-dependent efficacy of 2′-FL, 0.8% (*w*/*v*) was selected as the working concentration for all subsequent experiments.

To determine whether the observed effects of 2′-FL were attributable to growth inhibition, the growth curves of *F. nucleatum* were subsequently assessed (Figure S1). No significant effect of 2′-FL on bacterial growth was observed. To visualize the structural impact of 2′-FL on *F. nucleatum* biofilm formation, we performed crystal violet staining and scanning electron microscopy (SEM) following 48 h anaerobic incubation with 0.8% (*w*/*v*) 2′-FL. Under light microscopy, control biofilms exhibited dense, confluent purple staining across all magnifications, indicative of robust biomass accumulation and mature biofilm architecture (Figure 1c, left panels). In contrast, 2′-FL-treated biofilms appeared markedly sparse and discontinuous, with substantially diminished staining intensity and a loss of structural cohesion at 10×, 20×, and 40× magnifications (Figure 1c, right panels). Scanning electron microscopy (SEM) provided further ultrastructural evidence of the inhibitory effect of 2′-FL on biofilm formation. At 1000× magnification, control biofilms exhibited extensive surface coverage with a thick, interwoven meshwork of bacterial filaments, whereas 2′-FL-treated samples showed sparse bacterial colonization with large unoccupied surface areas. More strikingly, at 5000× magnification, untreated *F. nucleatum* cells displayed robust interbacterial adhesion, with elongated fusiform cells tightly juxtaposed and physically interconnected via multiple contact points, forming a cohesive three-dimensional biofilm matrix (Figure 1d). Conversely, in the 2′-FL-treated group, bacterial cells were predominantly dispersed as isolated entities or small, loose clusters, with a pronounced reduction in cell-to-cell contacts and interconnecting filaments. These morphological observations indicate that 2′-FL impairs the adhesive capacity of *F. nucleatum*, thereby compromising initial microcolony formation and subsequent maturation of the biofilm architecture.

### 3.2. 2′-FL Represses the Expression of Virulence Genes in F. nucleatum

**Figure 2:**
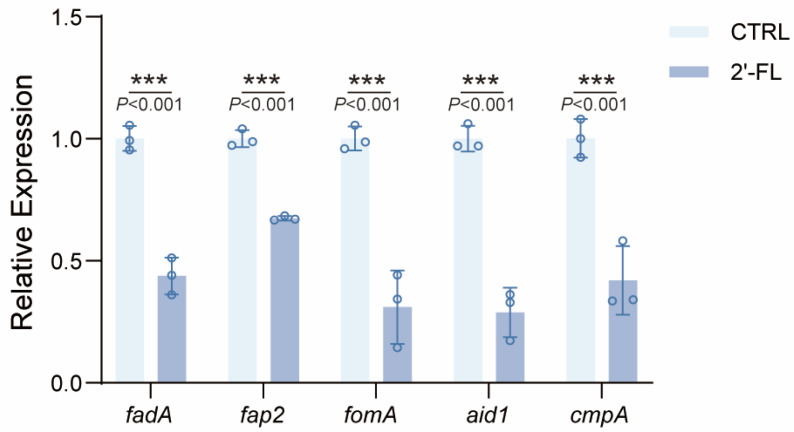
qRT-PCR analysis of *fadA*, *fap2*, *fomA*, *aid1* and *cmpA* gene expression in *F. nucleatum* following treatment with 0.8% (*w*/*v*) 2′-FL. Data was shown as the mean  ±  SD, n = 3. Differences between two groups were evaluated using an unpaired two-tailed Student’s *t* test. (*** *p*  <  0.001).

To elucidate the molecular mechanisms underlying the anti-biofilm activity of 2′-FL, we quantified the transcriptional levels of five key virulence genes—*fadA* (encoding a major adhesin), *fap2* (encoding a galactose-inhibitable adhesin), *fomA* and *cmpA* (encoding outer membrane proteins), and *aid1* (encoding an adhesion and invasion determinant)—in *F. nucleatum* following treatment with 0.8% (*w*/*v*) 2′-FL. As shown in Figure 2, qRT-PCR analysis revealed that 2′-FL significantly downregulated the expression of all five target genes compared with the untreated control (*p* < 0.001). Collectively, these findings indicate that 2′-FL modulates the expression of virulence-associated genes in *F. nucleatum*, thereby impairing bacterial auto-aggregation, epithelial adhesion, and biofilm maturation.

### 3.3. 2′-FL Reduces F. nucleatum Adhesion to Human Gingival Epithelial Cells

**Figure 3:**
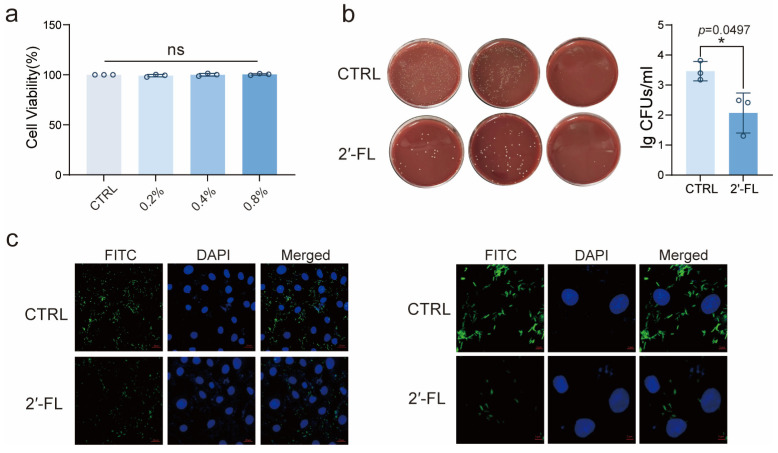
2′-FL attenuates *F. nucleatum* adhesion to human gingival epithelial cells (HGECs). (**a**) HGEC viability following 24 h exposure to 2′-FL at concentrations of 0.2% to 0.8% (*w*/*v*), determined by cell viability assay (n = 3). (**b**) Quantification of *F. nucleatum* adherent to HGECs by colony-forming unit (CFU) (n = 3), Left, representative images of anaerobic culture plates; right, bacterial counts expressed as log CFUs/mL. (**c**) Representative laser scanning confocal micrographs at magnifications of 40× and 120×, Statistical significance was determined by one-way ANOVA with Tukey’s multiple comparisons test. (* *p*  <  0.05, ns not significant.).

Prior to evaluating the anti-adhesive efficacy of 2′-FL against *F. nucleatum*, we first assessed its biosafety with human gingival epithelial cells (HGECs). As shown in Figure 3a, treatment with 2′-FL at concentrations ranging from 0.2% to 0.8% (*w*/*v*) for 24 h did not compromise HGEC viability, with cell survival rates exceeding 90% across all tested concentrations and no statistically significant difference relative to the untreated control. These findings establish 0.8% (*w*/*v*) as a safe working concentration for subsequent functional assays.

We next examined whether 2′-FL reduces the adhesive capacity of *F. nucleatum* to HGECs. As illustrated in Figure 3b, 2′-FL treatment resulted in a marked reduction in adherent bacteria compared with the control, with CFU counts decreasing by approximately 1.5 log units (*p* < 0.05), indicating that 2′-FL significantly suppresses *F. nucleatum* adhesion to gingival epithelium.

To visualize the spatial distribution of adherent bacteria at the cellular level, we employed laser scanning confocal microscopy following co-culture of FITC-labeled *F. nucleatum* with HGECs. In the untreated control group, confocal imaging at 40× and 120× magnifications revealed abundant green fluorescent bacterial cells densely clustered on and around HGECs, while DAPI-stained nuclei exhibited blue fluorescence. Numerous filamentous *F. nucleatum* cells were closely juxtaposed to the epithelial cell membrane, with occasional intracellular localization, consistent with the known invasive potential of this periodontal pathogen. In stark contrast, 2′-FL treatment markedly reduced the density of bacteria associated with the surface of HGECs, accompanied by a pronounced decrease in green fluorescence intensity at both magnifications (Figure 3c). These findings further confirm that 2′-FL substantially diminishes *F. nucleatum* adhesion to human gingival epithelial cells.

### 3.4. Molecular Docking of 2′-FL with F. nucleatum Adhesion-Associated Proteins

**Figure 4:**
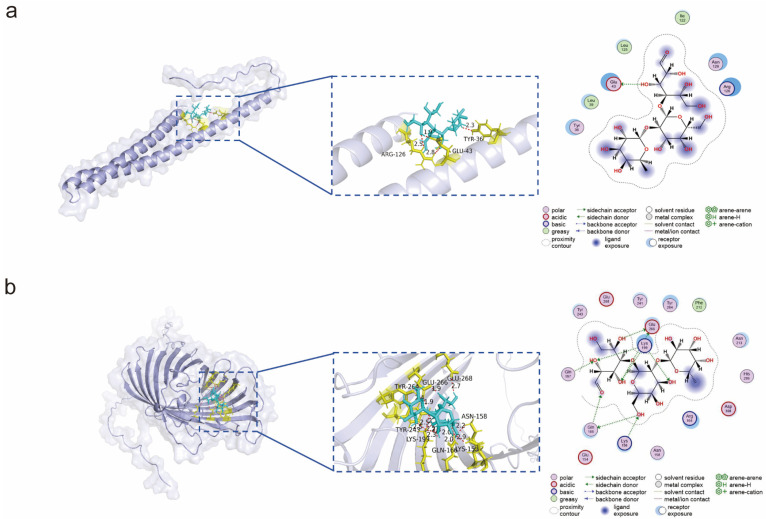
Molecular docking of 2′-FL with *F. nucleatum* virulence-associated proteins. (**a**) Docking of 2′-FL with the FadA adhesin. (**b**) Docking of 2′-FL with the FomA outer membrane porin.

To elucidate the structural basis underlying the anti-adhesion activity of 2′-FL, molecular docking simulations were performed between 2′-FL and two critical *F. nucleatum* biofilm-associated proteins, FadA and FomA. As illustrated in Figure 4a, docking of 2′-FL into the predicted binding region of FadA revealed favorable hydrogen-bond interactions between the O11 atom of 2′-FL and the carboxyl oxygen (OE1) of the Glu43 side chain, with a predicted bond distance of 2.84 Å and an interaction energy of −3.8 kcal/mol. The interatomic distance of less than 3.0 Å indicates favorable hydrogen-bond geometry, suggesting that this interaction may serve as an anchoring contact that stabilizes the orientation of the sugar ring within the FadA binding interface. Given that FadA is a principal adhesin mediating *F. nucleatum* attachment to and invasion of host epithelial cells, the predicted interaction between 2′-FL and the FadA suggests a potential mechanism by which 2′-FL may interfere with FadA-mediated bacterial adhesion.

Similarly, molecular docking of 2′-FL with the outer membrane porin FomA revealed an extensive hydrogen-bonding network comprising nine predicted interactions with key residues, including Glu266, Lys199, and Lys156 (Figure 4b). Notably, the hydrogen bond between ligand O11 of 2′-FL and Glu266-OE1 exhibited the most favorable predicted interaction energy (−4.7 kcal/mol), with a bond distance of 2.82 Å, suggesting that this residue may constitute a core anchoring site. Additionally, O13 simultaneously formed bidentate hydrogen bonds with Lys199 and Gln197, collectively contributing to the stabilization of the ligand–protein complex. These multiple predicted interactions positioned 2′-FL within the extracellular loop region of FomA, where it may interfere with FomA-associated adhesive functions. Collectively, these computational predictions suggest possible interactions between 2′-FL and adhesion-associated proteins, although experimental validation is required to confirm direct binding.

### 3.5. 2′-FL Protects Human Gingival Epithelial Cells from F. nucleatum-Induced Inflammation and Barrier Disruption

**Figure 5:**
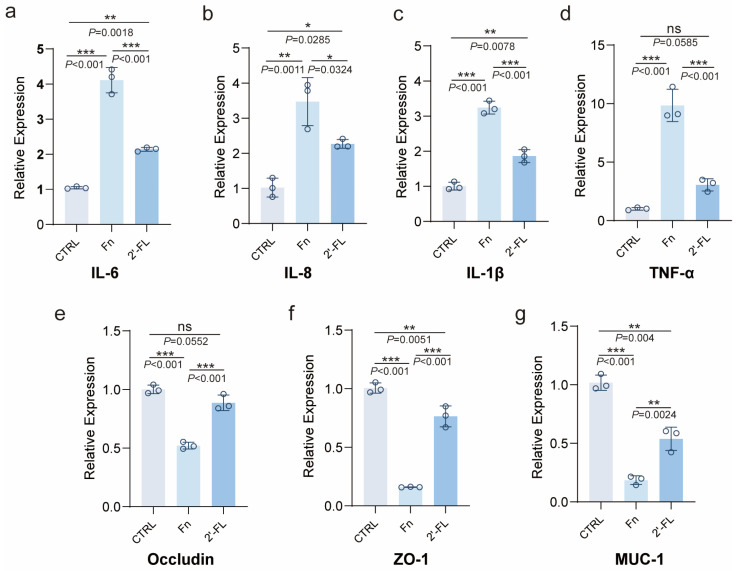
2′-FL attenuates *F. nucleatum*-induced pro-inflammatory cytokine production and preserves epithelial barrier integrity in human gingival epithelial cells. (**a**–**d**) qRT-PCR analysis of pro-inflammatory cytokine transcript levels, including (**a**) IL-6, (**b**) IL-8, (**c**) IL-1β, and (**d**) TNF-α, in HGECs following *F. nucleatum* infection (MOI =100, n = 3). (**e**–**g**) Expression of epithelial barrier-associated genes, including (**e**) Occludin, (**f**) ZO-1, and (**g**) MUC1, under identical experimental conditions, n = 3. Statistical significance was determined by one-way ANOVA with Tukey’s multiple comparisons test. (* *p*  <  0.05, ** *p*  <  0.01, *** *p*  <  0.001, ns not significant).

To determine whether 2′-FL attenuates the pro-inflammatory response elicited by *F. nucleatum* in gingival epithelium, we quantified the transcript levels of inflammatory mediator and barrier-related genes in HGECs following bacterial challenge. As shown in Figure 5a–d, at the transcript level, *F. nucleatum* infection markedly upregulated the expression of pro-inflammatory cytokines, including interleukin-6 (IL-6), IL-8, IL-1β, and tumor necrosis factor-α (TNF-α), compared with uninfected controls. Notably, 2′-FL intervention significantly suppressed the *F. nucleatum*-triggered elevation of all four cytokines. Concurrently, we assessed epithelial barrier-related gene expression by examining tight junction and mucin markers. *F. nucleatum* challenge significantly compromised barrier-associated marker expression in gingival epithelial cells, as evidenced by the downregulation of the tight junction markers occludin and zonula occludens-1 (ZO-1), as well as the transmembrane mucin MUC1 (Figure 5e–g). Remarkably, 2′-FL treatment substantially reversed these deleterious effects: occludin expression was restored to a level statistically indistinguishable from that in the uninfected control, whereas ZO-1 and MUC1 were significantly upregulated compared with the *F. nucleatum*-infected group, indicating partial restoration of barrier-associated markers. Collectively, these findings demonstrate that 2′-FL not only suppresses *F. nucleatum*-induced inflammatory signaling in gingival epithelial cells but also helps preserve epithelial barrier-associated gene expression, thereby attenuating periodontal pathogen-mediated epithelial damage.

### 3.6. 2′-FL Attenuates F. nucleatum-Induced Inflammatory Responses in Raw 264.7 Cells

**Figure 6:**
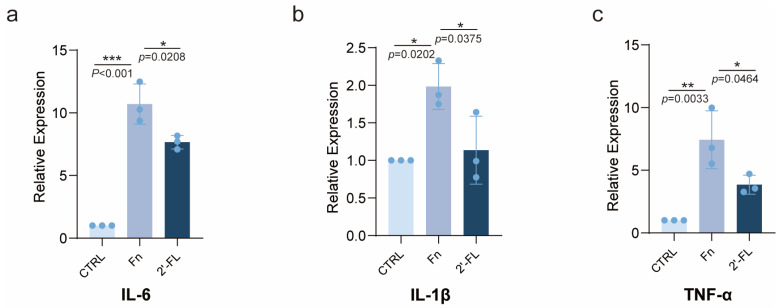
2′-FL suppresses *F. nucleatum*-induced pro-inflammatory cytokine expression in RAW264.7 macrophages. (**a**–**c**) qRT-PCR analysis of (**a**) IL-6, (**b**) IL-1β, and (**c**) TNF-α transcript levels in RAW264.7 cells following 24 h co-incubation with *F. nucleatum* (MOI = 100, n = 3) in the presence or absence of 0.8% (*w*/*v*) 2′-FL. Statistical significance was determined by one-way ANOVA with Tukey’s multiple comparisons test. (* *p*  <  0.05, ** *p*  <  0.01, *** *p*  <  0.001).

Previous studies have established that *F. nucleatum* activates the TLR4/NF-κB signaling axis to drive macrophage secretion of pro-inflammatory cytokines, including IL-6, IL-1β, and TNF-α, thereby promoting M1 macrophage polarization, exacerbating gingival inflammation, and ultimately triggering osteoclast differentiation and alveolar bone resorption [20]. To determine whether 2′-FL modulates this pathogenic cascade, we co-cultured RAW264.7 macrophages with *F. nucleatum* (MOI = 100) alone or in combination with 0.8% (*w*/*v*) 2′-FL for 24 h and quantified inflammatory transcript levels by quantitative real-time PCR. As shown in Figure 6a–c, *F. nucleatum* challenge markedly upregulated the expression of IL-6, IL-1β, and TNF-ɑ in RAW264.7 cells compared with uninfected controls. Notably, 2′-FL co-treatment significantly attenuated the *F. nucleatum*-triggered induction of all three cytokines. Collectively, these findings indicate that 2′-FL exerts a protective effect against the macrophage inflammatory response driven by *F. nucleatum*.

### 3.7. 2′-FL Attenuates F. nucleatum-Induced Periodontal Injury in BALB/c Mice

**Figure 7:**
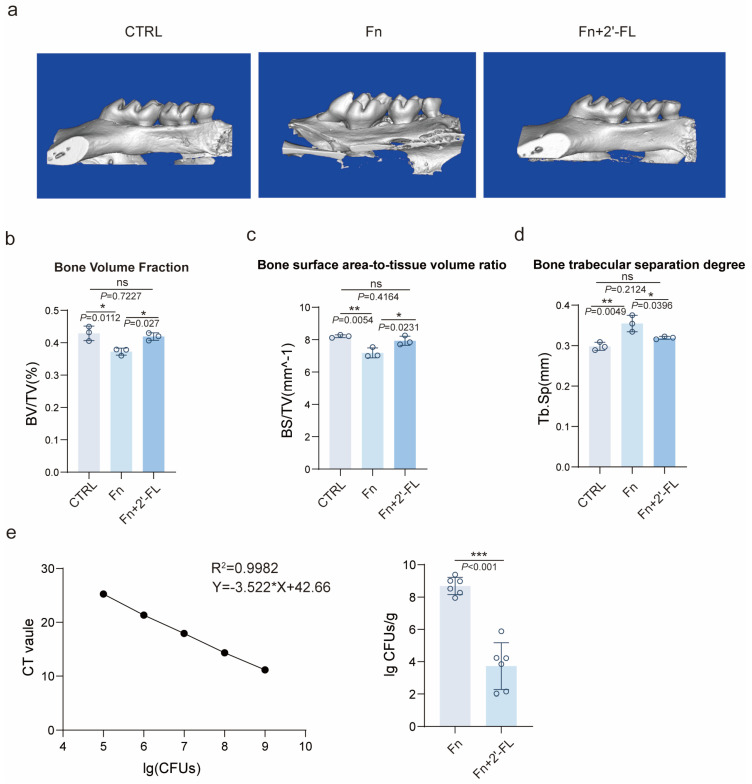
2′-FL attenuates *F. nucleatum*-induced alveolar bone loss and curtails bacterial colonization in balb/c mice. (**a**) Representative three-dimensional micro-computed tomography (micro-CT) reconstructions of maxillary alveolar bone in the intermolar region between the first and second molars. (**b**–**d**) Quantitative morphometric analyses of alveolar bone microarchitecture, including (**b**) bone volume fraction (BV/TV), (**c**) bone surface area-to-tissue volume ratio (BS/TV), and (**d**) trabecular separation (Tb.Sp). (**e**) Left, standard curve for *F. nucleatum* quantification by species-specific qRT-PCR (R^2^ = 0.9982); right, bacterial colonization burden in gingival tissue expressed as log colony-forming units per gram (lg CFUs/g), Data was shown as the mean  ±  SD (n = 3–6). Statistical significance was determined by Student’s *t* test and one-way ANOVA with Tukey’s multiple comparisons test. (* *p*  <  0.05, ** *p*  <  0.01, *** *p*  <  0.001, ns not significant.).

Micro-computed tomography (micro-CT) was utilized to evaluate alveolar bone microarchitecture in the intermolar region between the maxillary first and second molars. Three-dimensional reconstructions revealed pronounced alveolar bone resorption in *F. nucleatum*-infected animals, characterized by reduced trabecular bone mass and increased structural porosity, whereas 2′-FL treatment substantially preserved bone integrity (Figure 7a). Quantitative morphometric analysis confirmed that *F. nucleatum* infection significantly reduced the bone volume fraction (BV/TV) compared with uninfected controls, consistent with infection-associated alveolar bone loss (Figure 7b). Importantly, 2′-FL administration restored BV/TV to a level statistically indistinguishable from that of the control group. Furthermore, *F. nucleatum* challenge significantly altered the bone surface area-to-tissue volume ratio (BS/TV) and markedly elevated trabecular separation (Tb.Sp), reflecting deteriorated bone microarchitecture and enhanced bone resorption (Figure 7c,d). Strikingly, 2′-FL intervention reversed these pathological alterations: BS/TV was significantly increased compared with the *F. nucleatum* group and restored to baseline levels, while Tb.Sp was markedly reduced to values comparable to those in uninfected controls. To evaluate the inhibitory effect of 2′-FL on the colonization of *F. nucleatum* in vivo, we used species-specific primers to quantify bacterial burden by qRT-PCR. The standard curve showed excellent linearity (R^2^ = 0.9982), confirming the reliability of the assay (Figure 7e, left). Bacterial burden analysis revealed robust *F. nucleatum* colonization in infected mice, which was markedly suppressed following 2′-FL administration (Figure 7e, right). Histological examination further confirmed the protective effect of 2′-FL against periodontal inflammation (Figure S4). Healthy gingival tissues exhibited intact epithelial structures, densely packed collagen fibers, and minimal inflammatory infiltration. In contrast, *F. nucleatum* caused severe inflammatory cell accumulation, connective tissue edema, vascular dilation, and collagen fiber disorganization. Administration of 2′-FL markedly ameliorated these pathological alterations, as evidenced by reduced inflammatory infiltration, restoration of collagen organization, and improved gingival architecture, indicating effective attenuation of periodontal tissue damage. Collectively, these findings demonstrate that 2′-FL effectively attenuates *F. nucleatum*-induced periodontal injury and reduces bacterial colonization in vivo.

### 3.8. 2′-FL Modulates the Oral Microbiota Toward a Healthier Profile

**Figure 8:**
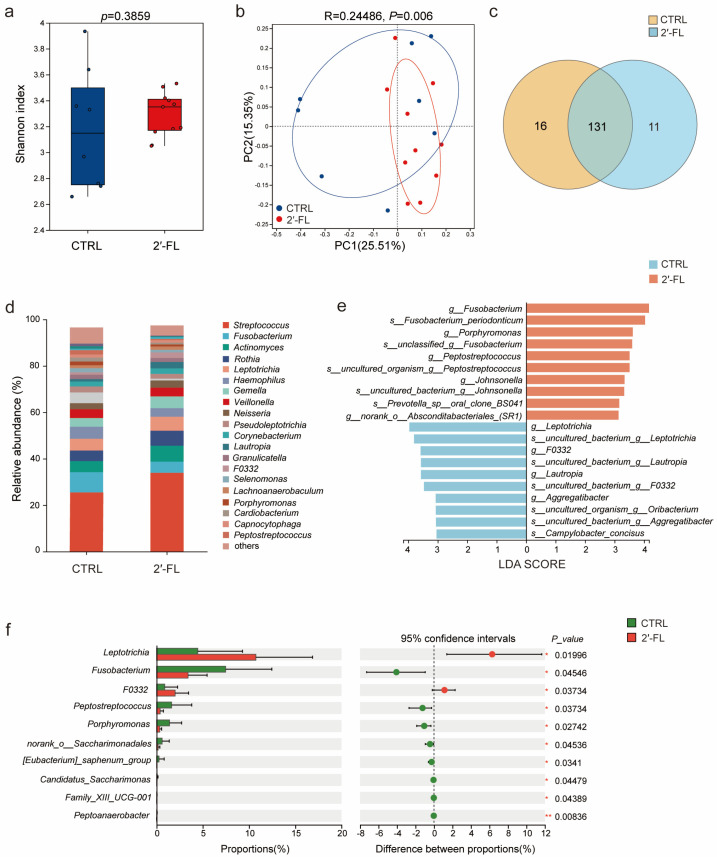
2′-FL reshapes oral microbial community structure. (**a**) Comparison of the Shannon diversity index between the CTRL (n = 8) and 2′-FL (n = 10) groups. (**b**) Principal coordinate analysis (PCoA) based on operational taxonomic unit (OTU)-level Bray–Curtis dissimilarities. (**c**) Venn diagram depicting shared and unique OTUs between the two groups. (**d**) Relative taxonomic abundance at the genus level. (**e**) Linear discriminant analysis effect size (LEfSe) bar plot showing differentially abundant taxa (|LDA score| ≥ 2.0) between groups. (**f**) Wilcoxon rank-sum test bar plot displaying differentially abundant genera, including proportions, 95% confidence intervals, *p*-values. Statistical significance was determined by two-tailed unpaired Student’s *t*-test, (* *p* < 0.05., ** *p* < 0.01).

To evaluate the modulatory effects of 2′-FL on the human oral microbiota, 16S rRNA sequencing was performed on oral plaque samples collected from healthy subjects following 2′-FL intervention. Alpha-diversity analysis revealed no significant difference in the Shannon diversity index and Chao index between the control and 2′-FL groups (Figure 8a and Figure S2). Principal coordinates analysis (PCoA) and non-metric multidimensional scaling (NDMS) based on OTU-level composition revealed a distinct clustering pattern between the two groups, suggesting that 2′-FL treatment reshaped the microbial community structure (Figure 8b and Figure S4). The Venn diagram analysis showed that there are 131 overlapping OTUs shared between the control and 2′-FL groups, while 18 and 11 OTUs were unique to the CTRL and 2′-FL groups (Figure 8c). Genus-level taxonomic composition analysis showed that the relative abundances of *Streptococcus*, *Actinomyces* and *Rothia* were increased in the 2′-FL-treated group, whereas the abundances of *Fusobacterium*, *Haemophilus* and *Porphyromonas* were reduced compared with those in the control group (Figure 8d). Subsequently, we conducted the linear discriminant analysis effect size (LEfSe) to discover the signature microbiota genera by assessing compositional differences between control and 2′-FL groups. At the genus level, the 2′-FL significantly enriched *Leptotrichia*, *F0332* and *Lautropia*, and the control group significantly enriched *Fusobacterium*, *Porphyromonas* and *Peptostreptococcus*; these differential bacterial genera serve as the major discriminatory taxa contributing to the microbial differences between the control and 2′-FL groups (Figure 8e). Consistent with the LEfSe results, Wilcoxon rank-sum analysis further confirmed that the relative abundances of *Fusobacterium* and *Porphyromonas* were significantly decreased in 2′-FL treatment group (Figure 8f). Notably, *Fusobacterium* and *Porphyromonas* are recognized periodontal disease-associated genera closely linked to oral dysbiosis and inflammatory tissue destruction [21]. Collectively, these findings indicate 2′-FL modulates the composition of oral microbiota by suppressing disease-associated taxa, and this is contributing to the restoration of oral microbial homeostasis and the maintenance of a healthier oral microenvironment.

## 4. Discussion

Oral health is a critical cornerstone of systemic well-being. Dysbiosis within the ecological niche of the oral microbiota not only precipitates localized pathologies, including dental caries and periodontitis, but also contributes to systemic inflammatory disorders [22,23]. *F. nucleatum*, an obligate anaerobic opportunistic pathogen, has emerged as a key orchestrator of oral microbiota dysbiosis, exerting profound disruptive effects on commensal community stability [24,25]. Traditional antimicrobial approaches for the management of oral infections have primarily relied on the use of broad-spectrum antibiotics to eliminate pathogenic microorganisms. However, such non-selective bactericidal strategies not only impose strong selective pressure that accelerates the emergence of antimicrobial resistance but also disrupt the ecological equilibrium of the resident oral microbiota, thereby exacerbating microbial dysbiosis [26]. Consequently, increasing attention has been directed toward the development of alternative therapeutic strategies based on natural bioactive compounds that can attenuate bacterial virulence and pathogenicity while simultaneously preserving or restoring microbial homeostasis. Such approaches are now regarded as a promising avenue for the prevention and treatment of oral infectious diseases. 2′-FL, the most abundant fucosylated HMO in human milk, has attracted considerable attention because of its ability to regulate host–microbe interactions, suppress inflammation, and selectively shape microbial ecology [27,28]. Accumulating evidence further indicates that the microbiota-modulating and immunoregulatory functions of 2′-FL extend beyond the gastrointestinal tract and may exert beneficial effects at extraintestinal sites [29]. In this study, we further explored the inhibitory mechanism of 2′-FL against the virulence of oral pathogenic bacterium *F. nucleatum* and evaluated its potential application in regulating oral microbiota. Our findings provide a scientific rationale for the incorporation of 2′-FL as a functional bioactive ingredient in next-generation oral healthcare products.

One of the most extensively studied functions of 2′-FL is its ability to inhibit bacterial adhesion and biofilm formation [30]. Due to its structural similarity to host cell-surface glycans, 2′-FL can function as a soluble decoy receptor that competitively interferes with bacterial attachment to epithelial cells. Previous studies have established that 2′-FL exerts anti-cariogenic and anti-adhesive activities against the oral cariogenic pathogen *Streptococcus mutans* [31]. Our findings further expand this observation, indicating that 2′-FL reduced the adhesion of *F. nucleatum* to HGECs. Molecular docking analysis provided a computational hypothesis regarding potential interactions between 2′-FL and FadA/FomA. However, these predicted interactions require further validation using biophysical approaches. Biofilm formation is a hallmark of periodontal pathogens and plays a central role in chronic oral infections by enhancing bacterial persistence, virulence, and resistance to host defenses [32]. Our research showed that, without affecting bacterial growth, 2′-FL effectively attenuated the virulence potential of *F. nucleatum* and inhibited its biofilm formation by downregulating the expression of key biofilm-associated virulence genes. Given that epithelial adhesion and biofilm formation represent critical initial steps in bacterial colonization and host invasion, these results suggested that 2′-FL may disrupt the pathogenic interaction between *F. nucleatum* and the host epithelium. In addition to its anti-adhesive and antibiofilm activities, 2′-FL has also been widely reported to possess anti-inflammatory properties. Previous investigations indicate that 2′-FL can suppress TLR4 expression and inhibit downstream NF-κB activation, thereby exerting anti-inflammatory effects [33,34]. *F. nucleatum* can adhere to and invade HGECs, where the FadA adhesin binds to E-cadherin on the cell surface, subsequently activating the TLR4/MyD88/NF-κB pathway to induce inflammatory cascades. Our findings demonstrated that 2′-FL reversed *F. nucleatum*-induced upregulation of *IL-6*, *IL-8*, *IL-1β*, and *TNF-ɑ* in HGECs, with comparable suppressive effects observed in RAW264.7 macrophages. We acknowledge as a limitation that these biological markers were evaluated exclusively at the transcriptional level via qRT-PCR, and without confirmation at the protein level. Finally, in vivo validation in BALB/c mice confirmed that 2′-FL mitigated *F. nucleatum*-induced periodontal tissue destruction and significantly reduced bacterial colonization burden. As excessive inflammatory signaling is closely coupled to osteoclastogenesis and alveolar bone loss, 2′-FL may preserve periodontal bone homeostasis by attenuating proinflammatory cytokine production and strengthening the gingival epithelial barrier.

Importantly, growing evidence has recently highlighted the role of 2′-FL in shaping microbial ecology. Unlike conventional antimicrobial preservatives that indiscriminately eliminate microorganisms, 2′-FL appears to selectively modulate microbial communities and promote ecological homeostasis. In the gastrointestinal tract, 2′-FL has been shown to enrich beneficial microorganisms, including *Bifidobacterium*, *Lactobacillus* and *Akkermansia muciniphila* [35,36,37], thereby enhancing mucosal barrier function and immune regulation [38]. However, the ecological effects of 2′-FL on the oral microbiota remain largely unexplored. In the present study, genus-level analysis revealed that 2′-FL treatment was associated with increased relative abundance of *Leptotrichia* and *Lautropia*, accompanied by reduced abundance of *Fusobacterium*, *Porphyromonas* and *Peptostreptococcus*. Both *Leptotrichia* and *Lautropia* are commonly recognized as health-associated members of the oral microbiota [39]. Previous microbiome studies have reported a higher abundance of *Lautropia* in caries-free individuals, suggesting a potential role in maintaining a health-associated oral ecosystem [40]. In contrast, members of the genera *Fusobacterium* and *Porphyromonas* are regarded as opportunistic pathogens and key contributors to oral microbial dysbiosis [41,42]. Under conditions that compromise oral ecological stability, these microorganisms can transition from commensal-like colonizers to pathogenic contributors [43,44]. Beyond their local effects within the oral cavity, these pathogens have also been implicated in the pathogenesis of a wide range of extraoral diseases through the oral–systemic axis, thereby linking oral dysbiosis to systemic health outcomes. Collectively, these findings suggest that 2′-FL functions as an ecological modulator of the oral microbiome, selectively suppressing opportunistic pathogenic taxa while helping preserve microbial equilibrium, thereby contributing to the maintenance of oral health and potentially mitigating the risk of dysbiosis-associated systemic diseases. But overall, our study is only a pilot study, and a larger clinical cohort is needed to validate the microbiota regulatory effect of 2′-FL.

Our findings provide a rationale for future studies investigating the feasibility of incorporating 2′-FL into mouthwash formulations. Conventional mouthwashes, particularly chlorhexidine-based formulations, are highly effective in reducing microbial burden; however, their long-term use is frequently associated with undesirable adverse effects, including tooth discoloration, taste perturbation, mucosal irritation, and disruption of oral microbial homeostasis [45,46]. Such limitations have prompted increasing interest in the development of alternative strategies that can selectively modulate pathogenic microorganisms while preserving the integrity of the resident microbiota. In contrast, 2′-FL is a naturally occurring and biocompatible oligosaccharide with a well-established safety profile, as evidenced by its widespread application in infant nutrition. Beyond its excellent biocompatibility, our findings suggest that 2′-FL possesses the capacity to attenuate pathogen-associated virulence while simultaneously promoting a balanced oral microbial ecosystem. Therefore, 2′-FL-based mouthwash formulations may represent a promising microbiome-friendly approach capable of suppressing pathogenic biofilm formation while maintaining oral microbial homeostasis, thereby potentially overcoming the limitations associated with conventional broad-spectrum antiseptic mouthwashes. Although the present study demonstrated the biological activity of 0.8% (*w*/*v*) 2′-FL under experimental conditions, the translational feasibility of achieving comparable concentrations in commercial mouthwash formulations warrants further consideration. Unlike systemic administration, mouthwashes are topically applied and are typically used in volumes of 10–20 mL for 30–60 s, making relatively high local concentrations technically achievable. Importantly, 2′-FL is highly water-soluble, chemically stable over a broad physiological pH range, and has an excellent safety profile established through its widespread use as a functional ingredient in infant formula [47,48]. These physicochemical and toxicological properties support its potential incorporation into oral care products. Nevertheless, successful clinical translation will require additional formulation studies to evaluate storage stability, compatibility with common mouthwash excipients (e.g., preservatives, flavoring agents, and buffering systems), retention within the oral cavity, sensory acceptability, manufacturing cost, and long-term safety and efficacy in human populations. Therefore, while the present findings provide a rationale for the future development of 2′-FL-containing mouthwash formulations, dedicated formulation optimization remains necessary before practical application.

## 5. Conclusions

In summary, our study demonstrated that 2′-FL attenuates virulence gene expression and biofilm formation in *F. nucleatum*. Moreover, 2′-FL suppresses *F. nucleatum*-induced inflammatory cytokine expression in human gingival epithelial cells and alleviates *F. nucleatum*-induced periodontal tissue damage in mice. In addition, 2′-FL modulates the oral microbial composition by reducing disease-associated taxa and promoting a more balanced oral microecological profile. Collectively, these findings expand the potential applications of 2′-FL in oral health management and provide a scientific rationale for future development and evaluation of 2′-FL-based oral care products.

## Data Availability

16S rRNA sequencing data and analysis codes are available in NCBI-SRA database (BioProject: PRJNA1475977).

## Supplementary Materials

The following supporting information can be downloaded at: https://www.mdpi.com/article/10.3390/microorganisms14071603/s1, Table S1: Primers for *F. nucleatum*; Table S2: Primers for Human gingival epithelial cells; Table S3: Primers for Macrophage (Raw264.7); Figure S1: Growth curves of *F. nucleatum* treated with 2′-FL; Figure S2: Histological appearance of gingival tissue after H&E staining; Figure S3: Chao index analysis between the control and 2′-FL groups; Figure S4: Non-metric multidimensional scaling (NDMS) analysis between the control and 2′-FL groups.
