# Supplementary material for: 2′-Fucosyllactose Attenuates Fusobacterium nucleatum Virulence and Modulates the Oral Microbiota
Source: Microorganisms. 2026 Jul 22;14(7):1603. doi: 10.3390/microorganisms14071603 (PMC13414073; doi:10.3390/microorganisms14071603)
Supplement: Supplementary file 1 [file microorganisms-14-01603-s001.zip › microorganisms-4415305-supplementary.pdf]

Figure S1: Growth curve of *F. nucleatum* treated with different concentrations of 2′-FL

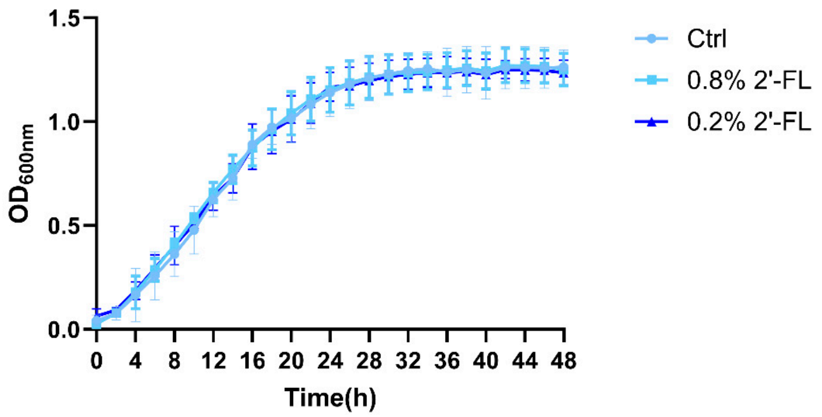

Figure S2: Histological appearance of gingival tissue after H&E staining. Representative H&E-stained gingival sections from the Control, *Fn*, and *Fn*+2'-FL treatment groups. Upper panels show low-magnification images, while the corresponding lower panels present higher magnification views of the gingival. *F. nucleatum* induced marked inflammatory cell infiltration, connective tissue edema, collagen fiber disorganization, and vascular dilation. Treatment with 2'-FL markedly reduced inflammatory infiltration and improved connective tissue architecture. Black arrows indicate loosened collagen fibers; Yellow arrows indicate inflammatory cell infiltration; Blue arrows indicate dilated blood vessels. Scale bars = 100 μm (upper panels) and 50 μm (lower panels).

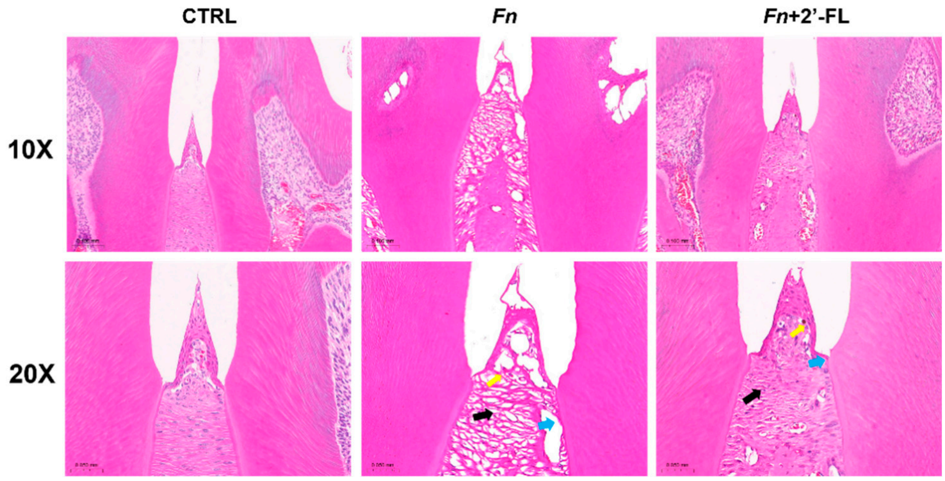

FigureS3: Chao index analysis between the control and 2'-FL groups.

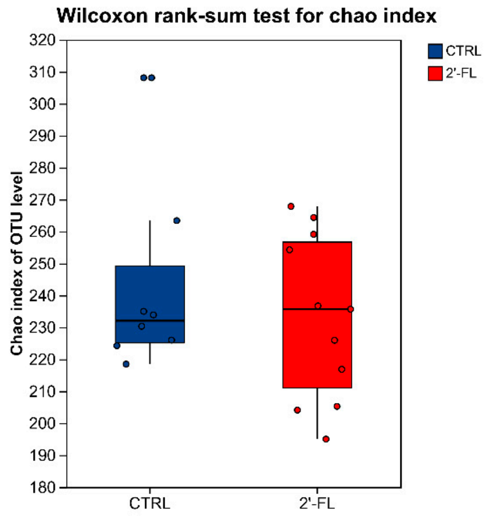

FigureS4: Non-metric multidimensional scaling (NDMS) analysis between the control and 2'-FL groups.

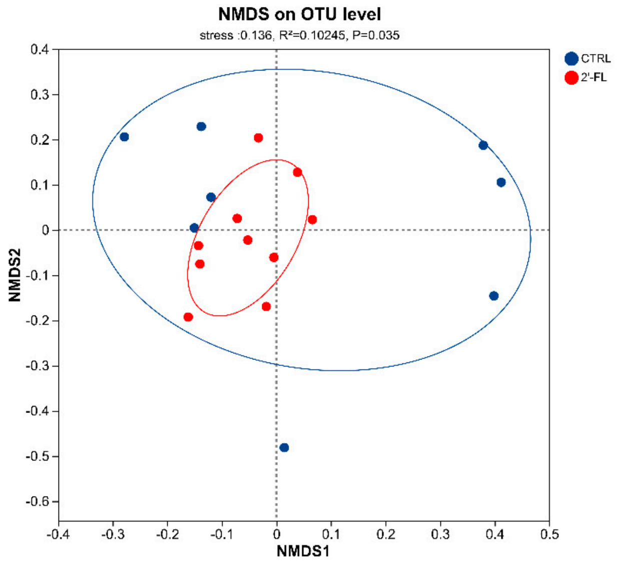

TableS1: Primers for *F. nucleatum*:

Primer	Sequence（5'-3'）
Fn-F	CAACCATTACTTTAACTCTACCATGTTCA
Fn-R	GTTGACTTTACAGAAGGAGATTATGTAAAAATC
FadA-F	TGCAGCAAGTTTAGTAGGTG
FadA-R	CATTGTAAACTTGTTCATTTTGT
Fap2-F	AAAATTGGAGCAACAGGAGGA
Fap2-R	TTCAGAGGCAATAGCGACAAC
FomA-F	AGAGTTTGATCCTGGCTCAG
FomA-R	GTCATCGTGCACACAGAATTGCTG
Aid1-F	TACAGGAGGTGCCGTAGCAG

Aid1-R	TTTTTGTTAATTCTCCAGCTCCA
CmpA-F	TTGGGATCAAGGAAAACATCAATTAGG
CmpA-R	ATAATTCCTTTATTATCTCCCATATAAGCAATACC

Table S2：Primers for Human gingival epithelial cells

Primer	Sequence（5’-3’）
IL-1β-F	CACGCTCCGGGACTCACAGC
IL-1β-R	CTGGCCGCCTTTGGTCCCTC
IL-6-F	AATCATCACTGGTCTTTTGGAG
IL-6-R	GCATTTGTGGTTGGGTCA
TNF-α-F	CCCAGGGACCTCTCTCTAATCA
TNF-α-R	GCTTGAGGGTTTGCTACAACATG
IL-8-F	CTTGGCAGCCTTCCTGATTTC
IL-8-R	CCAGACAGAGCTCTCTTCCAT
Gapdh-F	TTCACCACCATGGAGAAGGC
Gapdh-R	GGCATGGACTGTGGTCATGA
MUC1-F	TCCTTTCTCTGCCCAGTCTG
MUC1-R	GTGTGGTAGGTGGGGTACTC
OCLN-F	ACAAGCGGTTTTATCCAGAGTC
OCLN-R	GTCATCCACAGGCGAAGTTAAT
ZO-1-F	TGCCATTACACGGTCCTCTG
ZO-1-R	GGTTCTGCCTCATCATTTCCTC

Table S3: primer for *Raw264.7* Macrophage

Primer	Sequence（5’-3’）
IL-1β-F	GGGCCTCAAAGGAAAGAATCT
IL-1β-R	GAGGTGCTGATGTACCAGTTGG
IL-6-F	CTGGGAAATCGTGGAAATGAG
IL-6-R	AAGGACTCTGGCTTTGTCTTTCT
TNF-α-F	TCCCCAAAGGGATGAGAAGTT
TNF-α-R	GAGGAGGTTGACTTTCTCCTGG
Gapdh-F	TTCACCACCATGGAGAAGGC
Gapdh-R	GGCATGGACTGTGGTCATGA
